# The Effects of Auditory Contrast Tuning upon Speech Intelligibility

**DOI:** 10.3389/fpsyg.2016.01145

**Published:** 2016-08-09

**Authors:** Nathan J. Killian, Paul V. Watkins, Lisa S. Davidson, Dennis L. Barbour

**Affiliations:** ^1^Laboratory of Sensory Neuroscience and Neuroengineering, Department of Biomedical Engineering, Washington University in St. LouisSt. Louis, MO, USA; ^2^Central Institute for the Deaf, Washington University School of MedicineSt. Louis, MO, USA

**Keywords:** auditory cortex, noise reduction, human, cochlear implant, primate

## Abstract

We have previously identified neurons tuned to spectral contrast of wideband sounds in auditory cortex of awake marmoset monkeys. Because additive noise alters the spectral contrast of speech, contrast-tuned neurons, if present in human auditory cortex, may aid in extracting speech from noise. Given that this cortical function may be underdeveloped in individuals with sensorineural hearing loss, incorporating biologically-inspired algorithms into external signal processing devices could provide speech enhancement benefits to cochlear implantees. In this study we first constructed a computational signal processing algorithm to mimic auditory cortex contrast tuning. We then manipulated the shape of contrast channels and evaluated the intelligibility of reconstructed noisy speech using a metric to predict cochlear implant user perception. Candidate speech enhancement strategies were then tested in cochlear implantees with a hearing-in-noise test. Accentuation of intermediate contrast values or all contrast values improved computed intelligibility. Cochlear implant subjects showed significant improvement in noisy speech intelligibility with a contrast shaping procedure.

## Introduction

Hearing-impaired individuals in general, and cochlear implantees in particular, can often comprehend speech comparably to normal-hearing listeners in the absence of noise, but then exhibit poorer-than-normal performance in noisy environments (Friesen et al., 2001; Nie et al., 2005). In patients with sensorineural hearing loss, this outcome is due at least partly to the decreased perceived spectral contrast of speech sounds associated with diminished frequency selectivity in the auditory periphery (Leek et al., 1987; ter Keurs et al., 1993; Summers and Leek, 1994; Leek and Summers, 1996; Dreisbach et al., 2005). Spectral contrast represents the variation in sound energy distribution across frequency. Neuronal processing that utilizes spectral contrast to reduce noise is likely to be impaired in these individuals. Research into artificially compensating for spectral contrast deficits has resulted in a class of algorithms that perform a global spectral contrast enhancement. These algorithms represent signal processing strategies where peaks in the short-term signal spectrum are stretched relative to the troughs (i.e., the spectral variance is uniformly increased as a function of frequency). Previous studies evaluating the ability of spectral contrast enhancement to improve the intelligibility of noisy speech have demonstrated some potential benefit of this noise-reduction strategy for cochlear implant users and variable results for other subjects (Bunnell, 1990; Simpson et al., 1990; Stone and Moore, 1992; Baer et al., 1993; Ribic et al., 1996; Munoz et al., 1999; Loizou and Poroy, 2001; Lyzenga et al., 2002; Yang et al., 2003). Contrast enhancement likely achieves its effect by accentuating the information-carrying spectral peaks of vocalizations relative to the nearly uniform spectrum of stationary noise. Based upon our electrophysiological findings in the primary auditory cortex of a vocal primate species, we have developed a unique, biologically-inspired approach to compensate for deficits in spectral contrast processing.

**Figure 1:**
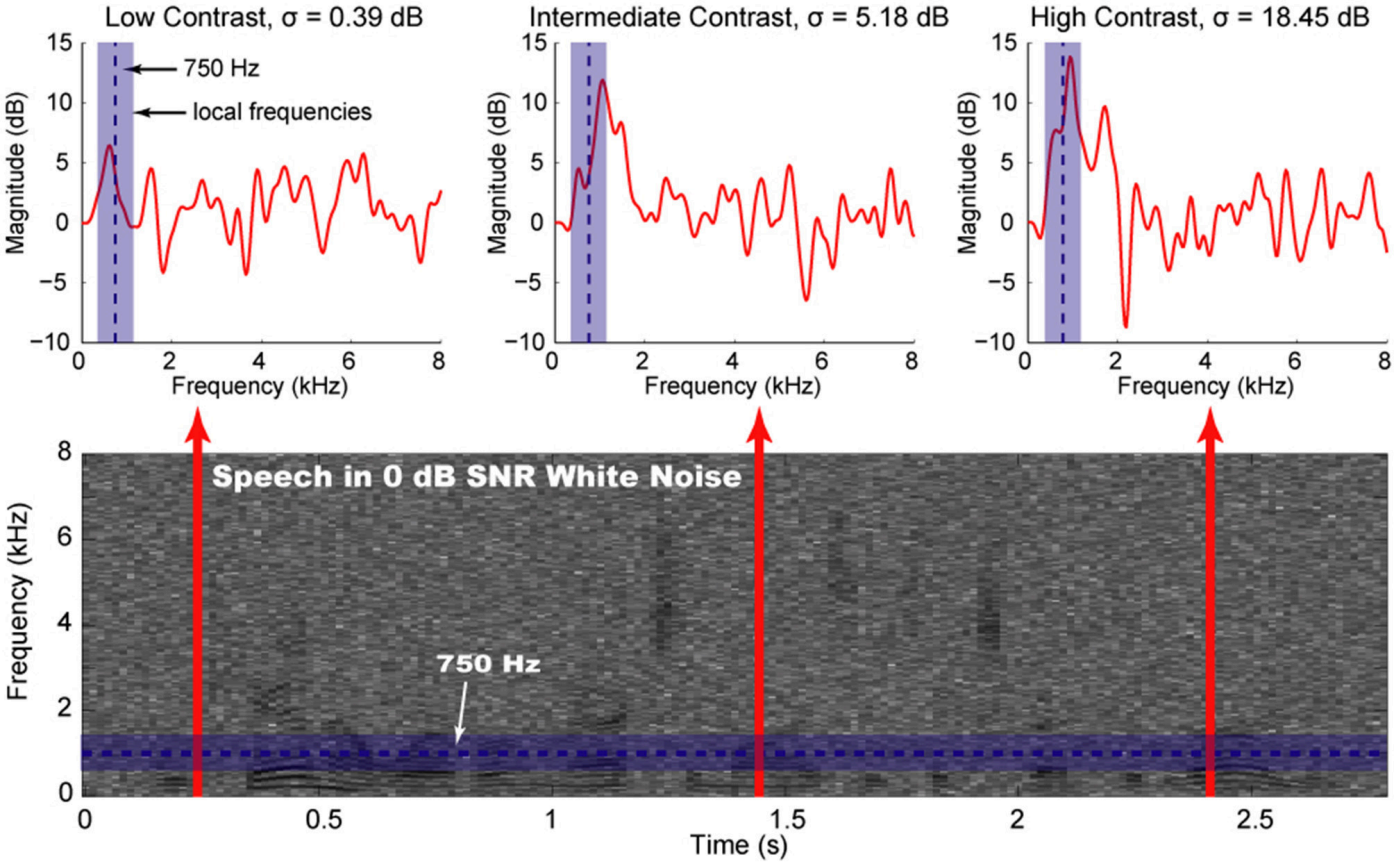
**Local contrast components of speech**. Shown are a spectrogram of a speech sample with white, Gaussian noise added (bottom) and magnitude spectra from three different time points (top). One frequency of interest (750 Hz, arbitrarily selected from frequencies containing speech) and the local frequency neighborhood (±400 Hz) are indicated with a dashed blue line and a blue rectangle, respectively. Spectral contrast is computed at a particular frequency by calculating the standard deviation (σ) of the spectrum for the local frequency neighborhood. Actual contrast calculations are made on filter outputs rather than spectrogram calculations. Low-contrast regions (left) tend to exhibit more noise than speech, while high-contrast regions (right) tend to exhibit more speech than noise. Intermediate-contrast regions (middle) are likely to contain speech and noise at similar powers.

We defined spectral contrast as the standard deviation of signal intensity in dB over short periods of time and relatively local ranges of frequency. Speech corrupted with additive white, Gaussian noise shows a considerable variety of spectral contrast values (Figure 1). Spectrotemporal signal regions of low contrast are likely to contain relatively more noise power than speech power; conversely, regions of high contrast are likely to have a greater contribution from the speech to the overall signal. Intermediate contrast values are characteristic of regions with a mixture of speech and noise at similar powers. Contrast enhancement algorithms do not explicitly compute spectral contrast and are thus applied uniformly to low-, intermediate-, and high-contrast regions. Emphasizing the low-contrast regions, however, would tend to accentuate the noise, while emphasizing the high-contrast regions would tend to distort the speech—either of which would likely decrease intelligibility. Furthermore, compressing low-contrast regions or preferentially emphasizing regions of intermediate contrast could aid a listener in discerning the differences between the noise and the speech, potentially leading to improved speech intelligibility. In this study, we expand upon previous contrast enhancement studies by systematically exploring the effects of enhancing or compressing specific contrast bands. We find that emphasizing high contrasts or low contrasts alone or in combination degrades the intelligibility of noisy speech in a variety of noise conditions. We also examine the effects of preferential emphasis of intermediate contrasts and enhancement of all contrast components.

Our motivation for decomposing stimulus contrast into separate bands comes from electrophysiological findings. We have identified neurons in the auditory cortex of a vocal primate species that encode spectral contrast non-linearly, such that their peak firing rate as a function of spectral contrast occurs at intermediate contrast values (Barbour and Wang, 2003). Among these relatively common contrast-tuned neurons, the peak of the distribution of preferred contrasts is in the range of 4–8 decibels of standard deviation (dB SD). Because regions of intermediate contrast represent a likely target for differentiating between speech and noise (Figure 1, middle), these neurons may be useful to the auditory system for extracting signal information (such as vocalization features) in the presence of noise. Based upon these neurophysiological findings, we created a computational signal processing array containing contrast-sensitive elements in order to mimic the observed neuronal contrast tuning. By analogy with the type of transformations in which contrast-tuned neurons appear to be involved, we then utilized this signal processing array to shape the spectral contrast of noisy speech. We evaluated the effects of this contrast shaping procedure on the perception of simulated cochlear implantees by calculating intelligibility of the processed speech relative to unprocessed speech. The quantitative measure we used to evaluate the result was shown previously to be appropriate for predicting noisy speech intelligibility following non-linear operations with both normal-hearing listeners and cochlear implant users as test subjects (Goldsworthy, 2005). The results indicated that contrast-shaping strategies may provide benefits to cochlear implant users listening to speech in noise. Noise-reduction strategies often show little benefit for non-implantees, even for individuals with considerable hearing loss (Kuk et al., 1990; Moore, 2003). Thus, we sought to test noise reduction in the population most likely to derive benefits: cochlear implantees. We tested one promising strategy by administering a hearing-in-noise test (HINT) to 5 cochlear implantees. Improved speech recognition in noise was seen, supporting the computational results.

## Materials and methods

Our methods can be broken down into distinct steps: (i) signal generation followed by preprocessing and application of contrast filters; (ii) weighting of the contrast filter outputs; (iii) reconstruction of the signal; and (iv) evaluation of the intelligibility of the reconstructed signal with either computational metrics or cochlear implantees. All computations in this study were performed using custom code written for MATLAB software.

### Signal generation

We obtained pre-recorded sentences spoken by native English speakers from the TIMIT speech corpus (used in computational analyses) and the HINT sets (used in both computational analyses and in cochlear implant testing) (Garofolo et al., 1993; Nilsson et al., 1994). The signals were degraded using additive noise that was pseudorandomly generated and scaled in the time-domain to be at the desired signal-to-noise ratio (SNR)—computed using the root mean square values of the separate speech and noise signals. The noise was separately computed to have desired characteristics and then added to the speech to create the sounds in the same form as they would be presented to a listener. Samples of noisy and processed noisy speech are included in the online Supplementary Materials. Sound levels in the supplementary audio files were normalized to the maximum level across all conditions at each SNR. Noise was generated by specifying the shape of the power spectrum up to the Nyquist frequency, combining this with phase values pseudorandomly sampled from a uniform distribution on 0 to 2π radians, making the frequency domain signal symmetric around the Nyquist frequency, and finally taking the inverse Fourier transform to obtain the noise in the time domain. White noise had a uniform power spectrum, pink noise had a 1/f spectrum, and spectrally-shaped noise had a spectrum that matched the speech. For the filter-bank processing, signals may be either normalized or un-normalized as it does not affect the computations performed on decibel values. In the cochlear implantee tests, no normalization was performed so that speech would be at the same level in the noisy and quiet conditions. However, to eliminate any bias due to changes in signal power after the noise-reduction strategies were applied, noisy speech that had processing applied was scaled to have the same overall signal power as the noisy speech without processing.

### Contrast-shaping algorithm and experimental design

**Figure 2:**
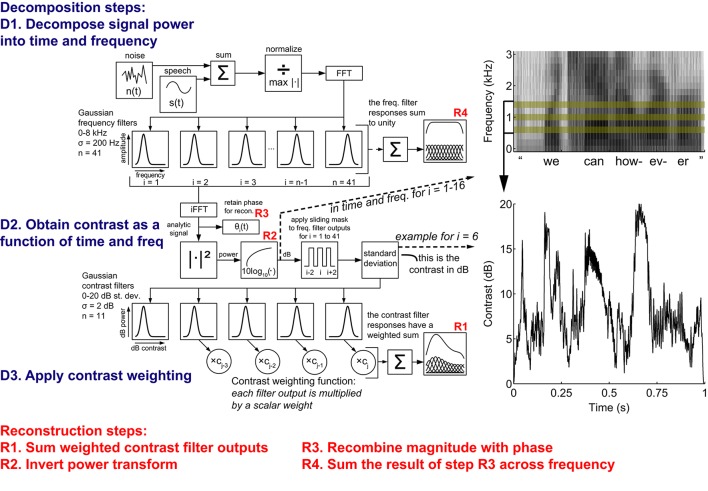
**A simplified schematic of the analysis portion of the local contrast shaping procedure**. The contrast shaping procedure is shown as a schematic and demonstrated on a sample utterance, “we can however.” For signals with additive noise, the frequency analysis sequence involves normalization followed by spectral filtering. The contrast analysis sequence involves obtaining the instantaneous signal power in dB, selecting the local frequencies of comparison (at the filter bank edges where *i* ∈ [1, 2, 40, 41], contrast is computed using 2 instead of 3 filters), calculating the standard deviation of the filter outputs at the local frequencies, and expanding/compressing the contrast based upon the contrast weighting function. On the top right is a time-frequency plot of the signal power (dB), showing only filters 1–16. The plot on the bottom right shows the local contrast of one region (SD in dB of 3 filters: 0.6, 1, 1.4 kHz) across time. The reconstruction portion reverses the analysis steps above (see Materials and Methods).

Contrast shaping was performed after filtering the noisy speech signals into different frequency (spectral) components and then into components associated with contrast across subsets of frequencies (spectral contrast). In other words, the contrast-shaping algorithm (Figure 2) contained two basic components of sound decomposition—frequency and contrast. Both of these components were designed to be reversible for reconstruction of the original time-domain signal. Both frequency and contrast decompositions were performed by filtering with an array of squared exponential, i.e., Gaussian, filters. For computational efficiency and so that the original signal could be reconstructed by summing the overlapping time segments, signals were windowed in time with 513-sample Hanning windows, overlapping by 257 samples. Sounds were sampled at 16 k samples/s, so that each time window duration was approximately 32 ms. Frequency and contrast decompositions were computed for each time window.

The frequency decomposition layer of the contrast-shaping algorithm partitioned the signal into linearly spaced frequency bands by transforming the time-domain signal into the frequency domain with a Fourier transform (using a Fast Fourier Transform algorithm) and multiplying the frequency-domain signal by the frequency response of each Gaussian frequency filter. These filters are given by Equation (1), where *m* is the number of frequency filters, the *X*_*i*_ are the frequency responses of the filters, and the μ_*i*_ are the means of the filters.

(1) $$\begin{array}{l} X_{i}\left(\omega \right)=e^{\frac{-{\left(\omega \;-\;\mu_{i}\right)}^{2}}{2\sigma^{2}}}\;\sigma \;=\;\mu_{i+1}\;-\;\mu_{i}\;i=1\ldots m \end{array}$$

Forty-one Gaussian frequency filters (σ = 200 Hz) linearly spaced from 0 to 8 kHz were used. The filters were equally spaced and the standard deviations set to the constant difference between the means so that the original signal could be reconstructed simply by summing the filter outputs (Theunissen and Doupe, 1998). We selected 41 linearly spaced Gaussian frequency filters empirically, as speech intelligibility scores for this arrangement were generally similar to or superior than intelligibility calculated when using other filter bank arrangements, such as logarithmically spaced triangular filters. The formulas presented in Equation (2) summarize the information retained from the frequency filters. The frequency response of each Gaussian filter was not symmetric, as the negative frequencies were set to zero. An inverse Fourier transform magnitude of each filter output is analogous to the amplitude envelope of that particular frequency band, and the power (squared amplitude envelope) of each of the 41 such signals, *f*_*i*_, was used as an eventual input into the contrast decomposition layer. The phase, θ_*i*_, of each analytic signal was retained for subsequent signal reconstruction. The input to the contrast layer was converted to dB power by applying Equation (3), yielding the *f_dB*_*i*_, which will be referred to collectively as the frequency bands.

(2) $$\begin{array}{lll} f_{i}\left(t\right) & = & |2FFT^{-1}\left(FFT\left(x\left(t\right)\right)X_{i}\left(t\right)\right){|}^{2}\\ \theta_{i}\left(t\right)\; & = & \;∠2FFT^{-1}\left(FFT\left(x\left(t\right)\right)X_{i}\left(t\right)\right) \end{array}$$

(3) $$\begin{array}{lll} f\text{\_}dB_{i}\left(t\right)\; & = & \;10\log_{10}\left(f_{i}\left(t\right)\right), \end{array}$$

The contrast decomposition layer of the contrast-shaping algorithm acted upon each frequency band and its neighboring frequencies. In other words, the local spectral contrast centered on each frequency band was calculated separately. Contrast was calculated as the standard deviation of a local subset of the frequency bands, which included the frequency band being decomposed and the second neighbor at both higher and lower frequencies, for a total of three frequency bands over which the standard deviation was computed. The equations in (4) describe this calculation, where the *c*_*i*_ are the contrasts centered at each frequency band, *m* is the number of frequency bands, *p* is the number of frequency bands used to compute local contrast, and the *f_dB*_*i*_ are the frequency bands themselves.

(4) $$\begin{array}{lll} c_{i}\left(t\right)\; & = & \;\sqrt{\frac{1}{p-1}\left(\underset{k}{\sum }{\left(f\text{\_}dB_{k}\left(t\right)-\bar{f\text{\_}dB_{i}\left(t\right)}\right)}^{2}\right)}\\ \bar{f\text{\_}dB_{i}\left(t\right)}\; & = & \;\frac{1}{p}\underset{k}{\sum }f\text{\_}dB_{k}\left(t\right) \end{array}$$

$$\begin{array}{lll} k\; & = & \;i\;-\;2,\;i,\;i\;+\;2\;p\;=\;3\;i\;=\;1\ldots m \end{array}$$

Although the subset of frequencies used to compute local contrast could be the focus of further investigations, preliminary results making use of different subsets (i.e., the local frequency mask in Figure 2, indexed by *k* in above equations) did not yield systematically improved intelligibility scores. These standard deviations were transformed by a set of Gaussian–shaped contrast channels linearly spaced in dB of standard deviation (dB SD), the units of our contrast measure. This Gaussian transformation described in Equation (5) shared the same form as the frequency filters described in Equation (1). Here *m* is the number of frequency bands, *n* is the number of contrast channels, the *S*_*i, j*_ are the contrast responses of each contrast channel (indexed by *j*) in each frequency band (indexed by *i*), and the μ_*j*_ are the means of the contrast channels.

(5) $$\begin{array}{lll} S_{i,\;j}\left(c_{i}\left(t\right)\right)\; & = & \;e^{\frac{-{\left(c_{i}\left(t\right)\;-\;\mu_{j}\right)}^{2}}{2\sigma^{2}}}\;\sigma \;=\;\mu_{j\;+\;1}\;-\;\mu_{j} \end{array}$$

$$\begin{array}{lll} j\; & = & \;1\ldots n\;i\;=\;1\ldots m \end{array}$$

Eleven contrast channels (σ = 2 dB SD) with means linearly spaced from 0 to 20 dB SD were used. As with the frequency filters, the contrast channels were equally spaced and the standard deviations set to the constant difference between the means so that the original signal could be reconstructed by summing the channel outputs (Theunissen and Doupe, 1998). Practical compromises with computational resources led to a maximum of 11 contrast channels. The outputs of each contrast channel *j* were then multiplied by the respective frequency band *i* (the dB power output from the respective frequency filter) to obtain a set of contrast channel outputs for each frequency band, *s*_*i, j*_ (Equation 6).

(6) $$\begin{array}{l} s_{i,\;j}\left(t\right)\;=\;f\text{\_}dB_{i}\left(t\right)S_{i,\;j}\left(t\right), \end{array}$$

The original time-domain signal was reconstructed by inverting the above steps for the contrast and frequency decompositions. Before reconstructing the original signal, we multiplied the contrast outputs by scalar weights, using a predetermined weight *w*_*j*_ for each contrast channel *j*. This step, given by Equation (7), where the *s*${}_{i,\;j}^{\prime }$ are the weighted outputs of the contrast channels, is referred to as the contrast-shaping step, and was added in order to emphasize or de-emphasize particular contrast ranges of interest.

(7) $$\begin{array}{l} s_{i,\;j}^{\prime }\left(t\right)\;=\;w_{j}s_{i,\;j}\left(t\right), \end{array}$$

The weights were applied directly to the log-transformed values, resulting in a non-linear effect. Because our contrast-shaping algorithm was invertible, if all contrast outputs received a unity weight, the reconstructed time-domain signal was virtually identical to the input signal. Non-unity weights, even when uniformly applied, have non-linear effects. These contrast-shaping manipulations would typically lead to an increase in local spectral contrast when a contrast channel was weighted > 1 and a decrease when a contrast channel was weighted < 1.

### Reconstructed signal metrics

**Figure 3:**
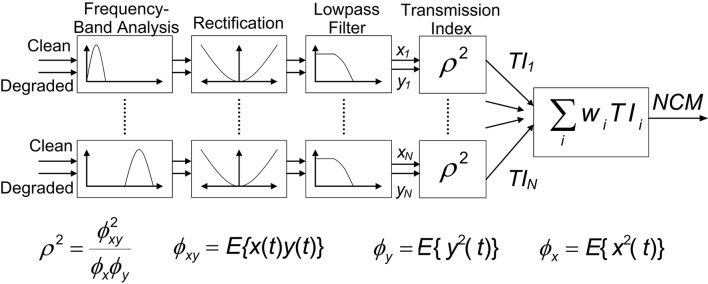
**The normalized correlation metric (NCM, adapted from Goldsworthy, 2005)**. The NCM computation begins with the calculation of Transmission Indices for each of *N* frequency bands as the normalized correlation between the two signals of interest (clean and degraded/unprocessed or clean and processed). A weighted sum of the normalized correlations for each frequency band produces the NCM (see Materials and Methods).

We related the intelligibility of the speech-in-noise (either before or after processing was applied) to clean speech using an approach that provides a measure of the correlation between the two signals. The normalized correlation metric (NCM) provided quantitative estimates of intelligibility of the processed signal (Goldsworthy, 2005). The NCM calculates a transmission index by a normalized correlation ρ^2^ between the envelopes of two signals (either clean and degraded signals or clean and processed) for a fixed number of frequency bands, *N*. Each of these *N* sub-band transmission indices is multiplied by a weight *w*_*i*_, determined by psychophysically evaluating the frequencies determined to be most important for speech intelligibility. Summing these values yields an NCM score between 0 and 1 (Figure 3). The NCM accounts for average envelope power in addition to temporal envelope fluctuations. It represents a metric empirically determined to be suitable for non-linear speech processing, such as in cochlear implants (Goldsworthy and Greenberg, 2004).

Mutual information (MI) (Cover and Thomas, 1991), specifically normalized MI (NMI) (Strehl and Ghosh, 2003), provided quantitative estimates of relative information content between noisy and clean speech independent of auditory system properties. Although contrast shaping cannot increase MI (as that would violate the data processing inequality, Cover and Thomas, 1991), MI is still informative because (i) finding changes analogous to NCM changes may support the generality of contrast shaping, and (ii) the results of contrast manipulations can be compared using the degree of signal degradation (the magnitude of the decrease in NMI after contrast shaping is applied). NMI was computed from Equation (8), where *X* and *Y* are the signals of interest (e.g., clean speech sample and contrast-shaped noisy speech), *p*(*x*) and *p*(*y*) are the amplitude histograms of the signals and *p*(*x*,*y*) is the joint amplitude histogram of the two signals. MI represents the sum of the marginal entropies of the signals, minus their joint entropy. While the MI is non-negative, it does not necessarily have an upper bound, making direct comparisons of information content among multiple experimental conditions challenging. The NMI, however, takes on values between 0 and 1, with a value of 0 for two statistically independent signals and a value of 1 for two identical signals. We selected this method of normalizing MI so that the metric is guaranteed to be upper bounded by 1, since the mutual information between a signal and itself is the entropy of the signal. Because the NMI uses each sample and the full range of each signal to estimate the probability distributions, sharp peaks of extreme high or low values may bias the result. To remove this bias, we used the signal envelopes at a resolution of *N* levels (i.e., the probability distributions were estimated with *N* bins).

(8) $$\begin{array}{lll} \text{MI}\left(X,\;Y\right)\; & = & \;\underset{y\in Y}{\sum }\underset{x\in X}{\sum }p\left(x,\;y\right)\log_{2}\frac{p\left(x,\;y\right)}{p\left(x\right)p\left(y\right)}\\ H\left(X\right)\; & = & \;-\underset{x\in X}{\sum }p\left(x\right)\log_{2}\;p\left(x\right) \end{array}$$

$$\begin{array}{lll} \text{NMI}\left(X,Y\right)\; & = & \;\frac{\text{MI}\left(X,Y\right)}{\sqrt{H\left(X\right)H\left(Y\right)}}, \end{array}$$

Both the NCM and NMI were computed with *N* = 24, unless otherwise indicated. The signal envelopes were low-pass filtered with a cutoff frequency of 50 Hz as an initial preprocessing step. Thus, for both metrics we used slow fluctuations in the signals as input, but the metrics differed in how they used these values; the NCM used the correlation of envelopes in *N* frequency bands, weighted based on their importance in human speech recognition, and the NMI used the input signals (here we used the broadband envelopes) at *N* level bins with no assumptions about which signal components are important for speech recognition.

### Systematic searches of the contrast weight space

We investigated the effects on speech intelligibility of performing manipulations such as enhancing or compressing distinct spectral contrast components. The possible weights on binned contrast (the contrast weight space) were explored in an unbiased, systematic manner. The contrast weight space was first explored in detail on a single TIMIT speech utterance (“We can, however, maximize the expected value”). Because the detailed search uses a great deal of computational resources, we performed this only on a single sentence. However, as drawing conclusions based upon this analysis alone might be confounded by overfitting, we then identified principal contrast regions of interest and evaluated them on a set of sentences from the HINT database.

The detailed systematic search of the contrast weight space was performed by iterating the contrast-shaping algorithm over all possible permutations of the weights 0.5 (compression), 1 (no change), and 2 (enhancement) for the 11 contrast channels (3^11^ weighting schemes). All weights were plotted as log base 2 values, falling within the range [−1 1]. Images of the systematic search results for the contrast weight space were created by sorting the contrast channel weight vectors by the computed NCM score. For visualization, these sorted weight vectors were then interpolated linearly in two dimensions at 1025 points uniformly spaced across each dimension width, between 0 and 20 dB contrast for the contrast weight dimension (abscissa) and between the minimum and maximum metric value for the metric dimension (ordinate). This 2D space was then smoothed with a Gaussian kernel whose standard deviations for both dimensions were set to 1/100 of the dimension range.

By visual examination of these images, specific contrast regions yielded clustered effects on intelligibility when weighting in a certain manner such as compression or enhancement. Based upon this analysis we used three contrast regions for subsequent analyses: low contrasts, the 0 and 2 dB bins; intermediate contrasts, the 4, 6, and 8 dB bins; and high contrasts, bins covering 10–20 dB. We again explored permutations of the logarithmically spaced weights on these three contrast regions (3^3^ = 27 weighting schemes). As this is computationally tractable, we performed this analysis on a set of 10 sentences from the HINT database (these sets were later used for testing with cochlear implantees).

### Psychophysical testing of algorithms in cochlear implantees

All human testing procedures were approved by the IRB at Washington University with written informed consent from all subjects. All subjects gave written informed consent in accordance with the Declaration of Helsinki. After providing written informed consent, five subjects with unilateral cochlear implants participated in this study. Etiology of hearing loss was most often reported as progressive (*n* = 3) with one subject experiencing a sudden hearing loss likely due to autoimmune disease and one subject reporting genetic hearing loss. All subjects used spoken English as their primary mode of communication. The mean length of implant use was 5.6 years and ranged from 2 to 11 years. The mean age at test was 69.4 years and ranged from 55 to 79 years. All subjects had full insertion of the electrode array and used a Cochlear Nucleus implant system (i.e., N22, N24). Speech recognition scores on the Consonant-Nucleus-Consonant (CNC) (Peterson and Lehiste, 1962) in quiet ranged from 43 to 84% correct. Demographic information for the subjects is provided in Table 1.

**Table 1:** **Subject demographic information**.

**Sex**	**Etiology**	**Age at study (years)**	**Length of CI use (years)**	**CI system**	**Processor**	**Implanted ear**	**CI coding strategy**	**CNC at test**
M	Progressive	57	2	Nucleus 24	Freedom	L	ACE	43
F	Progressive	79	5	Nucleus 24	Sprint	R	ACE	60
F	Sudden, unknown	77	6	Nucleus 24	Freedom	R	ACE	62
F	Progressive	79	4	Nucleus 24	Freedom	L	ACE	62
M	Genetic	55	11	Nucleus 22	3G	R	SPEAK	84

All testing was performed in a single-walled test booth typically used for clinical audiology research. Loudspeakers were positioned at 0° azimuth and placed one meter from the subjects. Calibration for the audiometer and binaural sound-field warble tones was conducted using a Type 1 sound level meter and ANSI Standards for Audiometers (ANSI S3.6-1996). All speech and noise stimuli were stored on a Dell Desktop computer. The computer was used to deliver the speech stimuli via an audiometer and loudspeaker. The HINT sentences were presented at a level of 65 dB SPL (slow RMS, C-weighting). The presentation level of the sentences was calibrated using a Type 2 sound level meter (Quest) and a 1000 Hz calibration tone.

Aided sound-field detection thresholds were obtained to assure audibility using frequency modulated (FM) stimuli at the following frequencies 250, 500, 1000, 2000, 3000, 4000, and 6000 Hz. A standard modified Hughson-Westlake procedure (Jerger et al., 1959) in increments of 2 dB was used to obtain thresholds. All subjects had aided thresholds at 30 dB HL or better from 250 to 6000 Hz.

Hearing in Noise Test (Nilsson et al., 1994) sentences were pre-recorded in the presence of stationary white noise at two signal-to-noise ratios (SNR, +5 and +10 dB). All sentences at each SNR were processed offline both with and without the noise-reduction strategy. Altogether, 16 sentence lists were used for the study with sentences within each list randomized across 5 test conditions (quiet, +5, +10 dB SNR unprocessed, and +5, +10 dB SNR with flat weights).

The HINT sentences were presented at an overall level of 65 dB SPL. Sentences in quiet and at each SNR for each condition (noise reduction/non-noise reduction) were presented randomly to each subject. All subjects used maps and processor settings (volume and sensitivity) that were worn in everyday listening conditions. After hearing each sentence, the subjects were asked to repeat the sentence back to the examiner. The number of words correctly repeated was recorded and the overall percent accuracy was each subject's speech recognition performance. Twenty sentences were presented at each of the five conditions for a total of 100 sentences. After a rest period, the subjects were presented with an independent set of 100 sentences. The NCM was used to predict performance using 260 sentences in the HINT database, comparing NCM^*^100 with percent correct speech recognition performance. This excludes prediction of quiet speech without additive noise or other processing where NCM = 1 (100% correct).

## Results

### Contrast shaping algorithm applied to noisy speech

**Figure 4:**
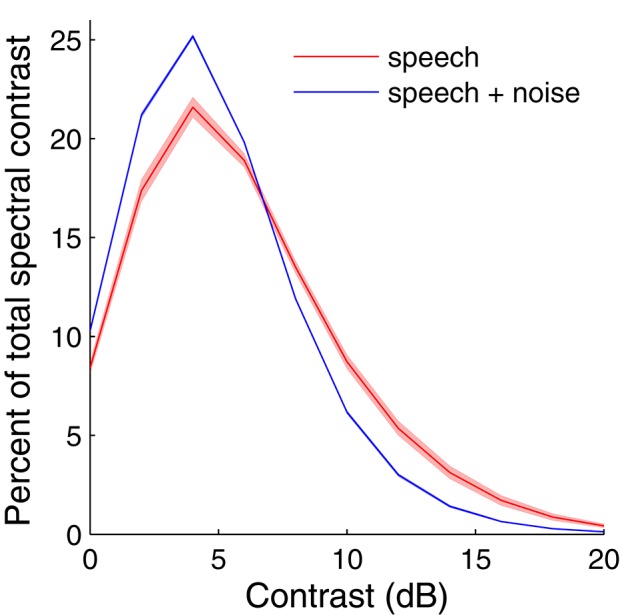
**Overlay of contrast distributions as calculated by the signal processing array for 10 sentences**. The overall spectral contrast distribution of a speech utterance indicates that most of the contrast values fall into the range of 4–8 dB. When the same speech utterance was corrupted with white, Gaussian noise at 5 dB SNR, the contrast distribution shifted to lower contrast values. Curves represent the mean across 10 sentences in the HINT database and the shading represents the mean ± standard deviation at each contrast value. The curves demonstrate that intermediate contrast values are common in both clean and noisy speech. Furthermore, there is little variability in contrast across sentences in the HINT database.

The overall spectral contrast distribution of the TIMIT speech utterance (“We can, however, maximize the expected value”), as determined by the output of the contrast elements in the signal processing array, peaked at around 5 dB SD of contrast (Figure 4). When white, Gaussian noise (0 dB SNR) was added, this curve adjusted toward lower contrast values, such that more portions of the noisy signal had lower contrast values and fewer had higher contrast values. The analytical portion of the signal processing array therefore outputs signal characteristics consistent with intuition and demonstrates that much of the dynamic range of local spectral contrast tends to be at intermediate contrast values. Subsets of trials run on other speech utterances and in other conditions verified the generality of the observations made with this speech sample.

We hypothesized based upon our previous neurophysiological findings that emphasizing mainly the intermediate contrast values could result in a substantial increase in speech intelligibility. To test this hypothesis, we modified noisy speech with the signal processing array. Signals were reconstructed from the outputs of 11 contrast-sensitive elements centered from 0 dB SD to 20 dB SD (see Materials and Methods). For a single noisy speech utterance, we systematically searched the space of all possible combinations of accentuation (log_2_(weight) > 0), no change (log_2_(weight) ≈ 0) and attenuation (log_2_(weight) < 0) of each contrast element output by measuring the NCM of the reconstructed signal as a computational estimate of speech intelligibility (see Materials and Methods).

**Figure 5:**
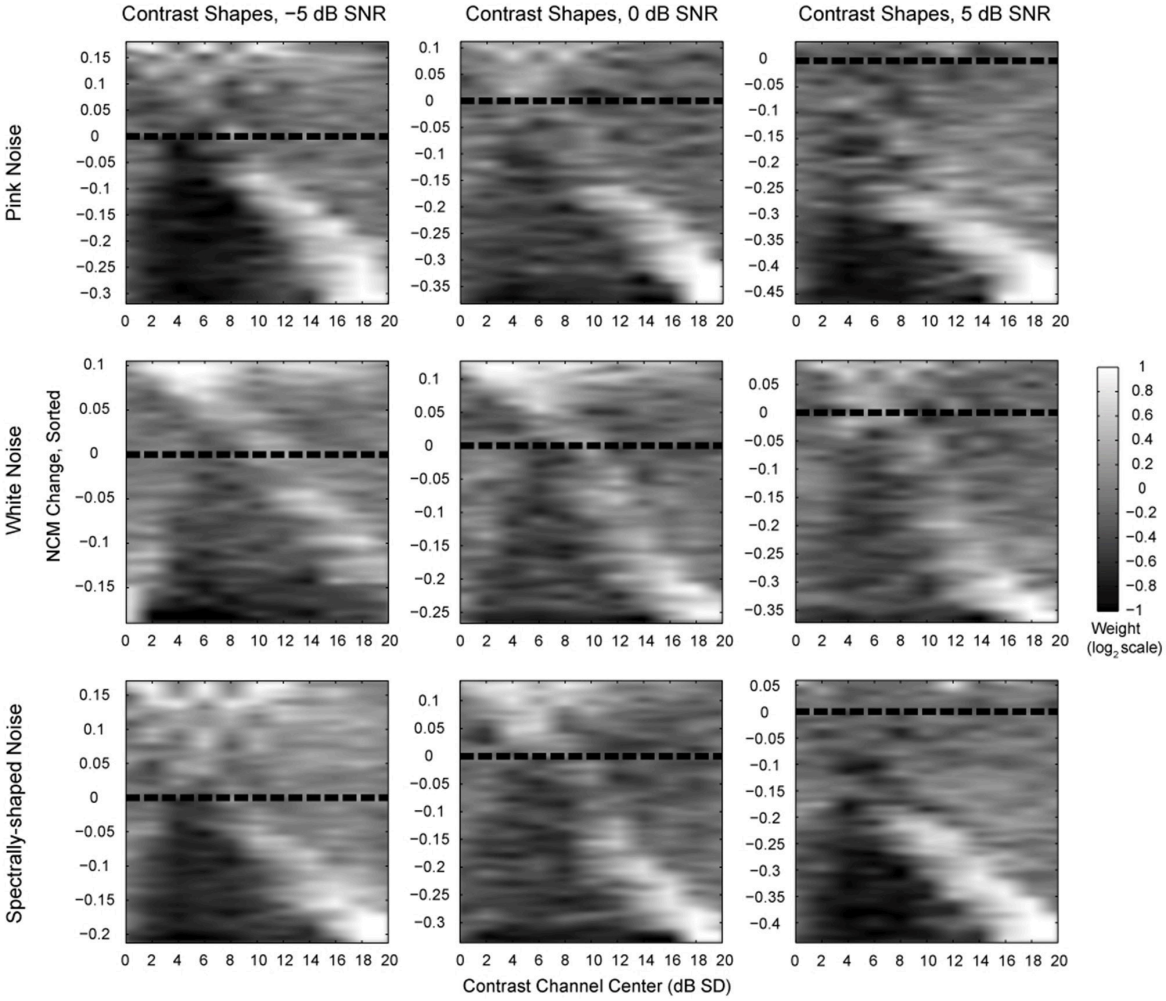
**The effects of various contrast weight vector shapes on noisy speech computed intelligibility**. Each panel represents a complete systematic search of the contrast weight space with different additive noise conditions and at different SNRs. The difference in NCM scores, calculated with *N* = 8 frequency bands, between the processed and original noisy speech for each contrast weight vector shape was used to sort the contrast shapes themselves. Each systematic search consisted of all permutations for weights of 2^−1^, 2^0^, and 2^1^ on each of the 11 contrast channels. The sorted contrast weights were then interpolated and smoothed to produce the display images. Lighter values correspond to higher weights, showing a convergence on intermediate contrast values at the best NCM scores. For pink noise, improvements achieved +0.04 (+5% relative to 0.87) at 5 dB SNR, +0.11 (+15% relative to 0.75) at 0 dB SNR and +0.18 (+30% relative to 0.6) at −5 dB SNR. For white, Gaussian noise, improvements in the NCM score achieved +0.09 (+12% relative to 0.77) at 5 dB SNR, +0.13 (+20% relative to 0.64) at 0 dB and +0.11 (+21% relative to 0.52) at −5 dB. For spectrally shaped noise, improvements achieved +0.06 (+7% relative to 0.84) at 5 dB SNR, +0.14 (+20% relative to 0.69) at 0 dB SNR and +0.17 (+33% relative to 0.51) at −5 dB SNR.

The patterns of contrast element weight vectors (i.e., contrast shapes) for the speech sample corrupted by pink noise (i.e., 1/f noise), white noise, and spectrally-shaped noise at three different signal-to-noise ratios (SNR) are shown in Figure 5. Contrast shapes in each panel are sorted by the change in NCM score of the processed noisy speech sample relative to the unprocessed noisy speech, yielding the most intelligible samples at the top of each panel and the least intelligible samples at the bottom. For the best improvements in computed intelligibility (top of each panel), modifying the highest and the lowest contrast values appears to influence the NCM scores little (i.e., mean weights near 0), while enhancement of the intermediate contrasts centered around 5 dB SD (i.e., weights near 1) appears to yield a substantial improvement in computed intelligibility. Enhancing the highest contrasts at the expense of the intermediate contrasts substantially worsens computed intelligibility scores (bottom of each panel). The same general patterns of contrast shaping functions for improving or degrading NCM scores are apparent across all types of noise and SNRs tested, as well as sample tests with other speech utterances (data not shown).

**Figure 6:**
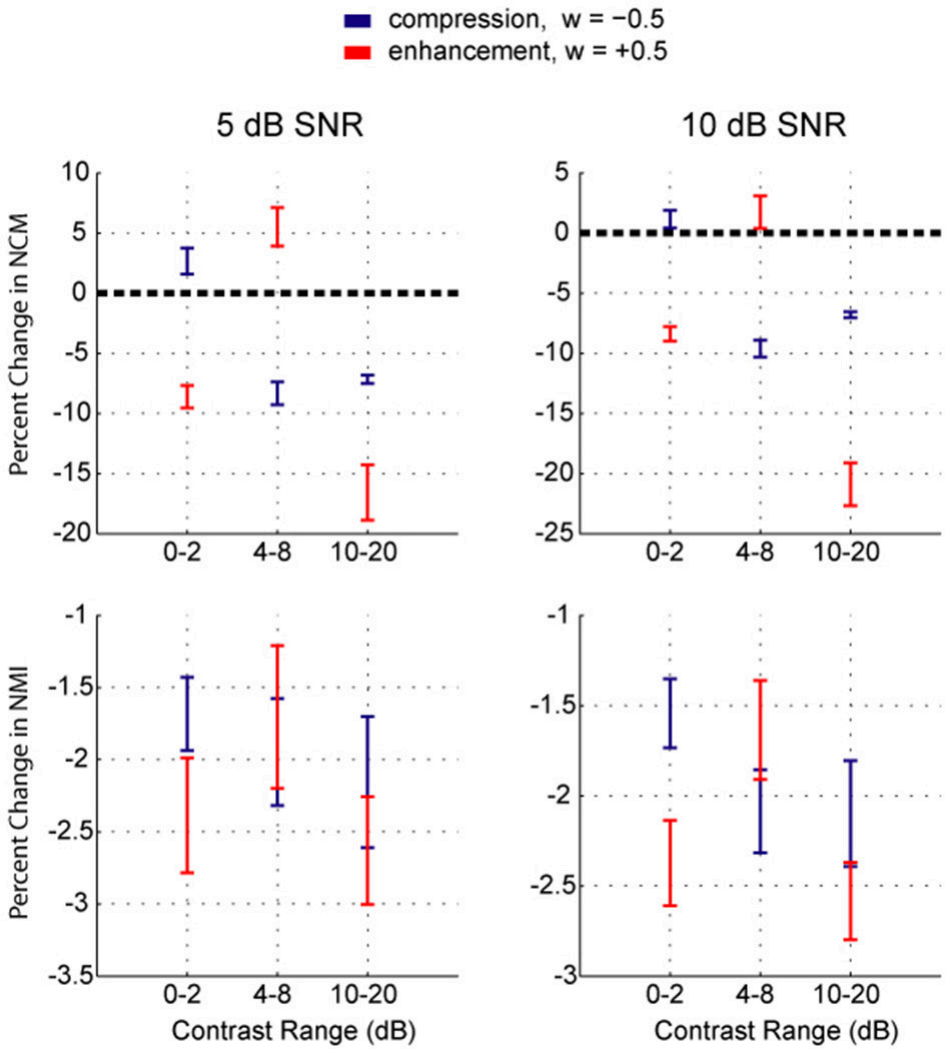
**Results of weightings applied to low, intermediate, and high contrast regions**. Either compression (−0.5) or enhancement (+0.5) weightings were applied to one of low, intermediate, or high contrast ranges of speech in Gaussian additive white noise at 10 dB SNR (left) and 5 dB SNR (right) (*n* = 10 sentences), with no weighting applied to the other ranges. The mean percent change in NCM and NMI scores is shown with error bars representing the SEM. The NCM scores show that enhancing low contrasts (mostly noise) or high contrasts (mostly speech) degraded intelligibility as predicted. Enhancing intermediate contrasts improved intelligibility on the average, as did compressing low contrasts. Compressing intermediate or high contrasts lowered intelligibility by about the same amount. The NMI scores showed similar trends, however the changes were more variable across the group of sentences. Note that because of the data processing inequality, the weightings cannot increase the mutual information.

The results shown in Figure 5 demonstrate some clustering in effects at roughly 0–2 dB, 4–8 dB, and 10–20 dB contrast ranges. To simplify the results shown in Figure 5, each of these three contrast bands was weighted independently of the others with either compression (weight of −0.5) or enhancement (weight of +0.5) and the resulting changes in the NCM and NMI scores were calculated. These data are displayed in Figure 6. As expected, compressing low contrasts or enhancing intermediate contrasts enhanced the predicted intelligibility. Expanding low or high contrasts strongly degraded performance.

**Figure 7:**
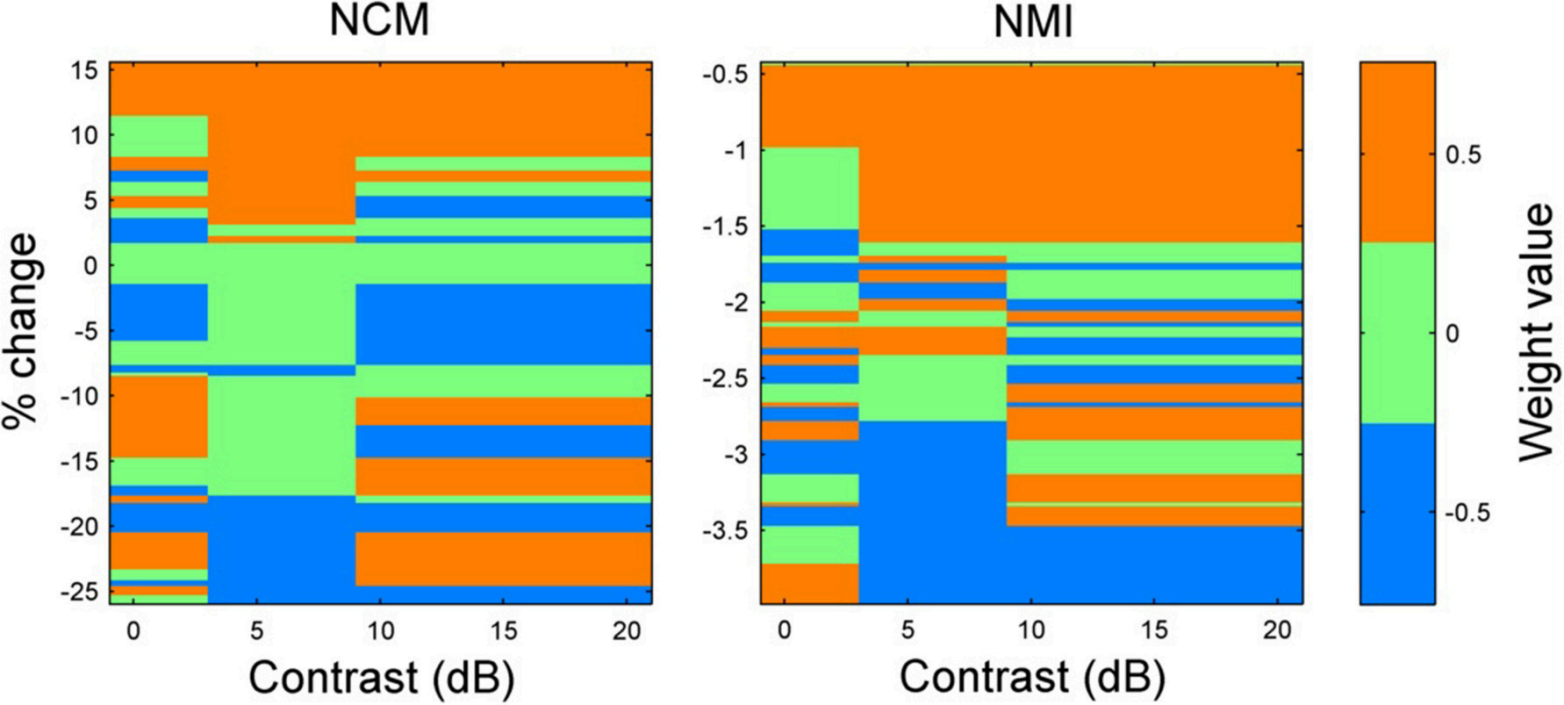
**Results of weights applied to low, intermediate, and high contrast bands**. Weights were applied to low, intermediate, and high contrast bands for 10 sentences with 5 dB additive white noise. The sorted averages of 10 sentences for the 27 weighting conditions are shown. The rows are centered on the actual value on the vertical axis and the row heights have been scaled to split the distance between successive weighting conditions. The worst NCM and NMI scores were obtained when performing simultaneous compression of intermediate and high contrasts. Importantly, any enhancement of low contrasts or high contrasts when intermediate contrasts were not also enhanced, lowered the NCM score. Furthermore, any compression of intermediate contrasts lowered the NCM score. The optimal weighting scheme for both the NCM and NMI scores (ignoring the case of no weighting for the NMI) is uniform enhancement.

When ranking the intelligibility scores for all permutations of the weights −0.5, 0, and 0.5 applied to these three bands, it becomes clear that enhancement of intermediate contrasts is generally beneficial regardless of the weighting applied to low and high contrasts (Figure 7). Furthermore, enhancing low or high contrasts is only beneficial when intermediate contrasts are also enhanced. Interestingly, if taking a linear summation approach, the results presented in Figure 6 suggest that low compression, intermediate enhancement, and no change to high contrasts would be beneficial. While beneficial relative to no enhancement, this strategy gives on average the 8th best NCM and NMI scores (out of 27 weighting schemes). It is actually the case of enhancing all contrasts that is the optimal weighting scheme. Thus, some uniform skewing of the contrast distribution to the right produces a signal that most closely approaches the original speech signal.

**Figure 8:**
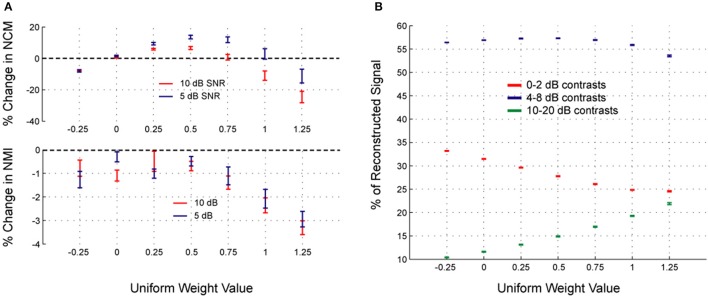
**Changes in signal metrics and contrast proportions after contrast shaping**. The changes in the signal metrics after a range of uniform contrast weightings were applied are shown for 10 sentences with additive white noise at 5 or 10 dB SNR. In terms of the percent change in the NCM, uniform compression (weight < 0) always degraded intelligibility, while uniform enhancement (weight > 0) enhanced intelligibility up to about a weight of 0.75, beyond which further enhancement (larger weights) degraded intelligibility (**A**, top). The percent change in NMI is relatively more variable, but demonstrates analogous properties in terms of degradation (i.e., enhancement degrades the signal less than compression, up to at least a weight of 0.5; **A**, bottom). The optimal weight (as indicated by the NCM scores, since any processing degrades the NMI) was on average 0.5 for the 10 sentences tested (**A**, top). The optimal weight coincided with the maximum percentage of intermediate contrast values in the signal **(B)**. For all plots, data points are presented as the sample mean ± SEM (*n* = 10).

**Figure 9:**
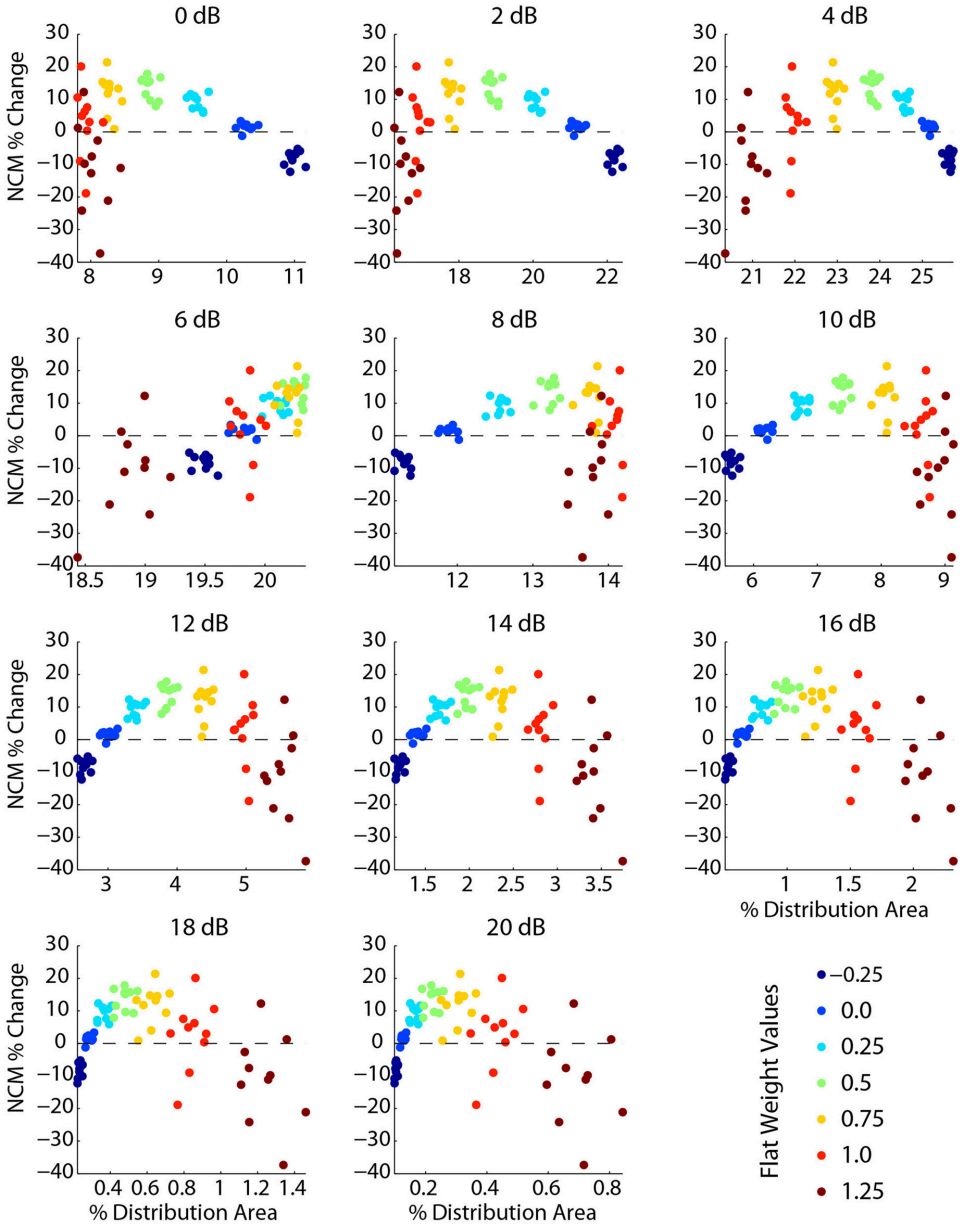
**Intelligibility is significantly correlated with the fractional amount of intermediate contrast in the signal**. Each plot shows the percent change in NCM score vs. the percent of signal content (percent of histogram area) for one of the 11 contrast bins. Each point represents one of 10 sentences (additive Gaussian white noise, 5 dB SNR) and one flat contrast weighting. The color of each data point represents the weight level (blue->red corresponds to −0.25 to 1.25, green is the optimal weight of 0.5). The 6 dB contrast bin has a strong positive correlation of intelligibility to fractional signal content. Larger weights lead to less fractional signal content at contrasts below 6 dB and more content for contrasts above 6 dB, reflecting that positive contrast weighting skews the spectral contrast distribution toward higher contrasts.

**Figure 10:**
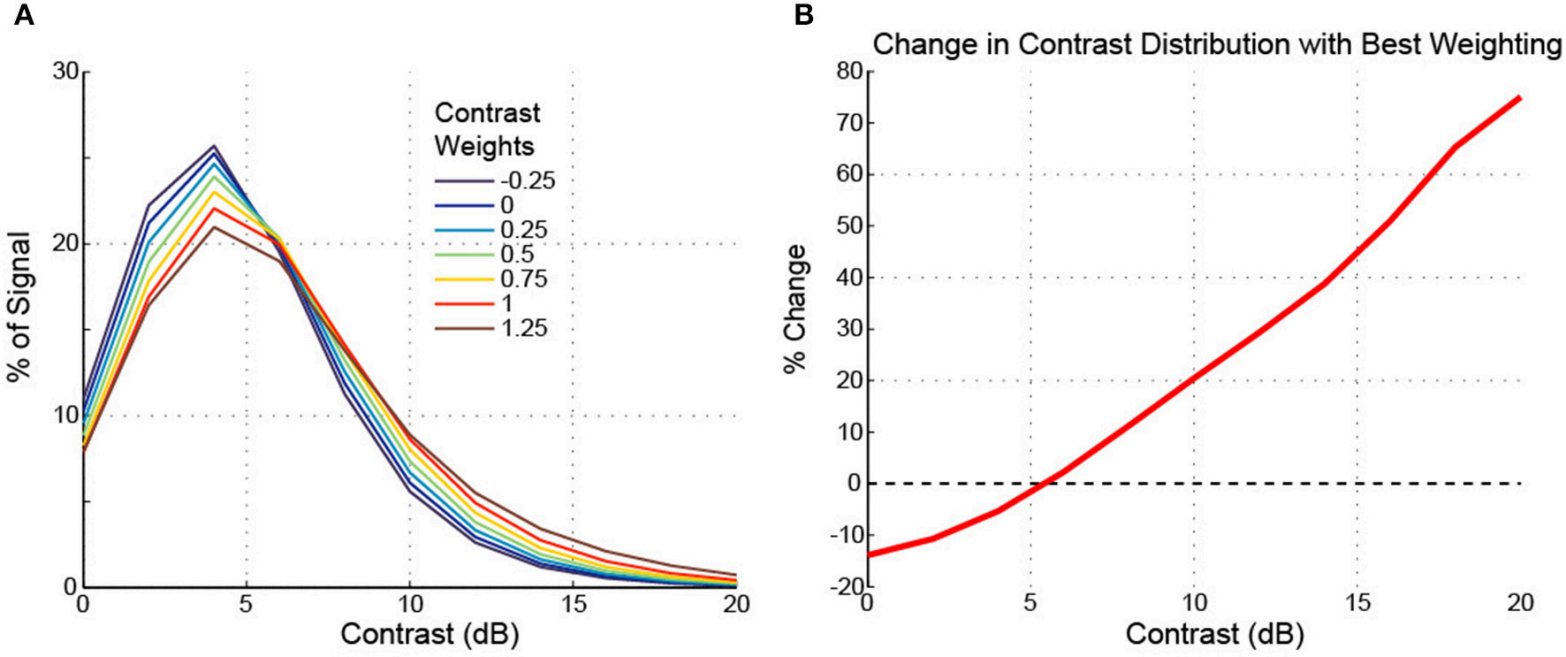
**Uniform weights and changes in the contrast distribution**. Increasing/decreasing the flat weight value skews the contrast distribution toward the right/left **(A)**, with very little change near 5–6 dB **(B)**. As seen in **(B)**, with the best weighting (0.5), there is a pivot or “isosbestic” point around 5–6 dB. In a roughly linear manner, the relative amount of lower contrasts decreases and the relative amount of higher contrasts increases. In comparison to Figures 8, 9, while the fraction of intermediate contrast energy is linearly correlated with intelligibility, the percent change possible is relatively small. The data shown are for one sentence, but the trends were nearly identical for all sentences tested.

To further examine the effects of flat/uniform weightings, weights from −0.25 to 1.25 were applied to noisy speech (Figure 8). For the sentences tested, the optimal weight (greatest relative increase in NCM score, Figure 8A, top) was 0.5, which coincided with optimum intermediate contrast area (Figure 8B). The NMI was also computed (Figure 8A, bottom) and showed analogous effects: enhancement degraded the signal less than compression up to an optimal weight, and larger weights degraded the signal more. Figure 8B shows that as the uniform weight value was increased, the amount of low contrast decreased, the amount of high contrast increased, and the amount of intermediate contrast showed a maximum at the optimal uniform weight value. The results shown in Figure 8 suggest that the fractional amount of different contrast regions might correlate with the intelligibility. Indeed, the fractional amount of contrast at 6 dB had a strong positive correlation with the NCM score (Figure 9). Other intermediate contrast values had weak positive correlations, while low and high contrast values had negative correlations (with the slight positive correlation at 10 dB being the only exception). Interestingly, this significant correlation between fractional intermediate contrast composition and intelligibility was concurrent with relatively little change in the distribution near 6 dB for any of the flat weightings (Figures 9, 10B). Increasing (decreasing) uniform weights resulted in right (left) shifting of the contrast distribution for all sentences tested (Figure 10A). At the best weighting (+0.5), the greatest proportional changes in the distribution (relative to no weighting) are at low and high contrasts, with little change near intermediate contrasts (Figure 10B).

**Figure 11:**
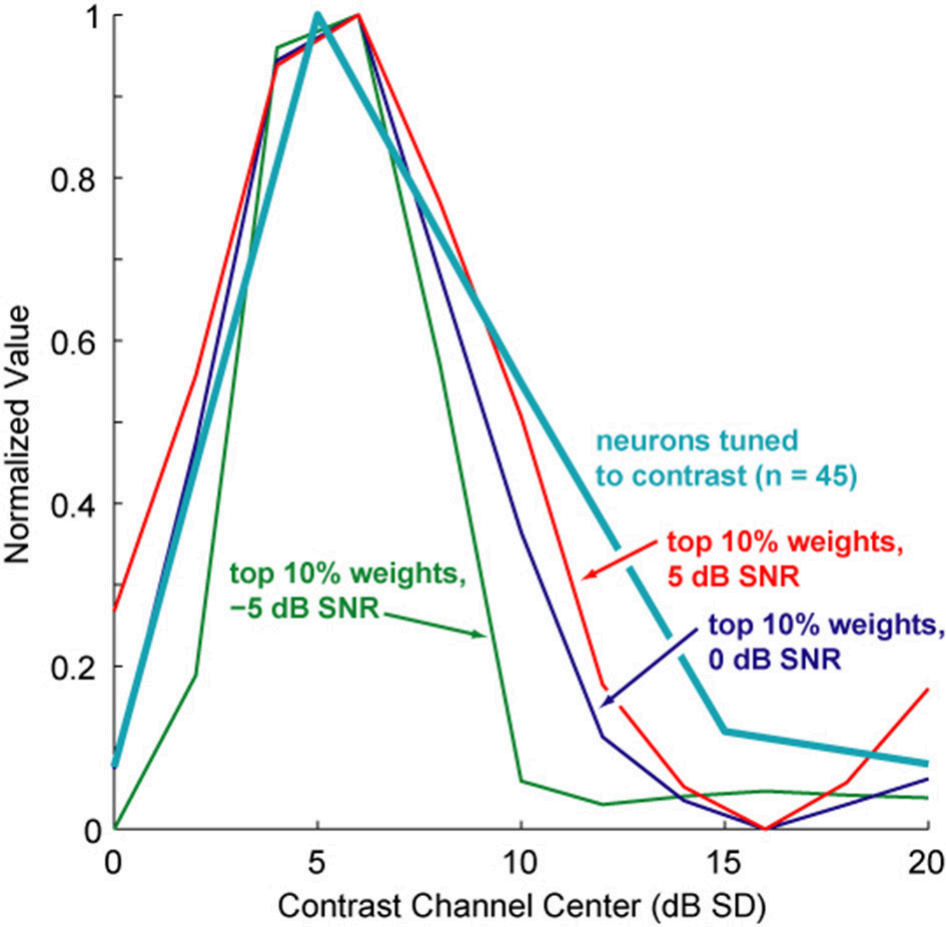
**Comparison of best contrast weighting shapes to preferred contrasts of contrast-tuned neurons**. Shown is an overlay of averaged contrast shapes achieving the best computed speech intelligibility improvements (from the systematic weight search, see Figure 5) compared with the distribution of peak contrast responses from a population of 45 contrast-tuned auditory cortex neurons. The normalized shapes of the best 10% of contrast shapes for 5, 0, and −5 dB SNR of white, Gaussian noise closely resemble this neuronal response distribution. Additionally, these contrast weighting distributions resemble one another even more, each one peaking at around 5 dB SD, despite a total SNR range of 10 dB across all three signals.

To evaluate how these computational results compared with neurophysiological data, we compared the average contrast shapes that elicited the greatest increase in NCM scores to the neuronal contrast data. Figure 11 shows the top 10% of NCM score improvements for the speech sample corrupted with white noise at three different SNRs. The shapes of these curves generally match the similar curves from other noise conditions. Overlaid on these plots is the distribution of preferred contrasts across the population of contrast-tuned neurons in primate auditory cortex (Barbour and Wang, 2003). The largest percentage of contrast-tuned neurons prefers contrasts that are also the most relevant, as determined by our contrast-shaping algorithm, for improving the intelligibility of noisy speech. Note that even though a total range of 10 dB SNR is represented in the speech signals tested, the best contrast shapes in all cases tended to peak at around 5 dB SD. The major difference between the best contrast shapes for different noise conditions appears to be predominantly the total range of contrast values emphasized rather than a shift in their peaks (i.e., lower noise levels lead to a broadened range around the peak at 5 dB). This similarity in shape underscores our empirical observation that contrast shaping can successfully improve computed intelligibility without any estimate of signal or noise properties.

### Evaluation of contrast shaping effectiveness in cochlear implantees

**Figure 12:**
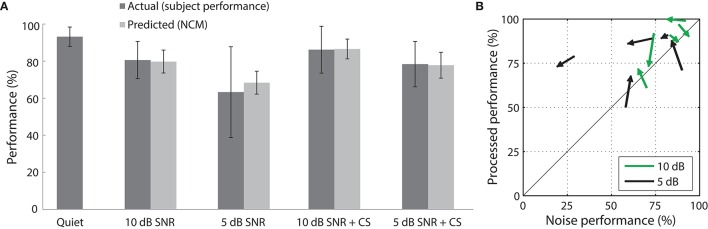
**Speech recognition performance of cochlear implant subjects evaluated with the HINT in 5 conditions. (A)** Displayed are the raw performance scores (mean ± standard deviation) of cochlear implantees (*n* = 5 subjects) and the predicted performance (mean ± standard deviation; *n* = 260 sentences). Improved speech recognition was seen in the 5 and 10 dB SNR flat weighting conditions. **(B)** Recognition performance (% correct) with contrast shaping is plotted against performance on speech in noise without processing (% correct) for each test session (*n* = 5 subjects, 2 test sessions per subject). The arrows point toward the scores from the second test session.

To evaluate the effectiveness of our contrast filter bank and weighting methods, we tested the best weighting scheme from simulations (+0.5 uniform weight) on 5 cochlear implant users. The subjects exhibited systematically degraded speech recognition performance as noise was added in a hearing-in-noise test (Figure 12). Contrast shaping that uniformly accentuated all contrasts between 0 dB and 20 dB (i.e., with a flat contrast shaping function) resulted in considerable predicted and actual intelligibility improvement (10 dB SNR: +7% predicted, +6% actual; 5 dB SNR: +9% predicted, +15% actual). Predicted performance was within 1% of actual performance on average for processed speech in noise and within 10% on average for noisy speech. The average improvement relative to the performance on speech in noise was greater for the 5 dB SNR condition (24%) than for the 10 dB condition (7%). The variable effects of degradation by the two SNR levels and performance of individual subjects on both test sessions are depicted in Figure 12B.

Hearing performance was analyzed with logistic regression using the following predictors: noise (5 or 10 dB SNR), contrast shaping, and test order. By performing a chi-squared test on the reduction in deviance with the inclusion of each predictor, we found that lower SNR was correlated with decreased performance [χ^2^(1) = 16, *p* = 5.9 × 10^−5^], as expected. There was no effect of the first or second set of sentences on performance [χ^2^(1) = 0.19, *p* = 0.67], suggesting that subjects did not have any learned changes in their ability to decode speech in noise over the course of the experiment. Finally, as predicted, contrast shaping was correlated with improved speech recognition performance [χ^2^(1) = 11, *p* = 8.4 × 10^−4^]. To examine the interaction between processing and noise level, the product of values representing noise level and contrast shaping was included as a predictor. The inclusion of this additional predictor did not result in statistically significant improvements in the model fit [χ^2^(1) = 1.4, *p* = 0.23], but noise level and contrast shaping remained significant predictors (*p* < 0.05). Given the small sample size of five cochlear implant users, further verification of these results with a larger sample of cochlear implantees is necessary.

## Discussion

### Contrast shaping algorithm applied to noisy speech

The contrast distribution of clean speech, as determined by the outputs of the contrast elements in our contrast-shaping algorithm, peaks at intermediate contrast values (Figure 4). This peak shifts toward lower contrast values when noise is added to the speech. If contrast-tuned neurons do aid in coding vocalizations or other sounds in the presence of noise, one might therefore expect that intermediate contrast values around 5 dB (the peak in the contrast-tuned neuronal population) could be particularly important for speech intelligibility. Stemming from recognition that lower spectral contrast results in lowered speech intelligibility, contrast enhancement algorithms attempt to shift the contrast distribution of noisy speech toward higher contrasts. This procedure can yield a higher proportion of higher contrast regions in the enhanced noisy signal—a result that tends to improve speech intelligibility. Using our contrast-shaping algorithm we found that a substantial increase in computed noisy speech intelligibility can potentially be obtained by accentuating primarily the intermediate contrasts (4–8 dB SD) (Figures 5–7). Accentuating the lowest contrasts (< 4 dB SD) or the highest contrasts (>8 dB SD) alone consistently degraded computed intelligibility. Compression of low contrasts enhanced computed intelligibility whereas compression of intermediate or high contrasts degraded intelligibility. On average, applying a uniform enhancement yields the best computed intelligibility (Figure 7). Interestingly, over a range of uniform weighting schemes, the fractional amount of intermediate contrast in the signal is simultaneously linearly correlated with computed intelligibility (Figure 9) and changes relatively little across that range (Figures 9, 10). Thus, the optimal contrast-shaping strategy decreases the relative amounts of low contrast components, and increases the relevant amounts of intermediate and high contrast components. These trends were consistent for the different noise types and SNRs tested. The greatest magnitude of computed intelligibility improvements occurred at the lowest SNRs tested, implying that contrast manipulations may be more effective for improving speech intelligibility under conditions of greater noisy interference. While we did not identify a significant interaction between SNR and contrast shaping, the results from testing in cochlear implantees are consistent with the computational findings. Cochlear implantees performed relatively better when processing was applied to 5 dB SNR speech in noise compared to 10 dB SNR speech in noise (24% vs. 7% improvement), with one patient consistently having a three-fold improvement at 5 dB compared to a more modest improvement at 10 dB (see Figure 12B).

### The role of contrast-tuned neurons

The effects of contrast shaping on a computed intelligibility score, the NCM, indicated that the contrast shapes most effective at improving computed speech intelligibility on average closely matched the distribution of best contrasts in the auditory cortex of marmoset monkeys (Figure 11). Furthermore, the peaks of the contrast weighting functions fell near 5 dB for a range of SNR values, indicating that a similar noise-reduction procedure might be effective across a variety of SNRs. We computed the intelligibility of the processed speech at many combinations of accentuation and attenuation of contrast, sorting by intelligibility score and taking the mean of the best contrast-weighting functions. The resulting functions compared favorably with the representations of contrast-tuned neurons in primate auditory cortex. These computational data were derived from an algorithm designed to improve computed speech intelligibility and not by fitting physiological data. This independent result lends plausibility to the hypothesis that contrast-tuned neurons may participate in a biological contrast-shaping algorithm whose purpose is to extract vocalizations or other sounds from a noisy environment. While no causal relationship has yet been established between contrast-tuned neurons and the effect of contrast shaping on altering computed speech intelligibility as reported here, this scenario represents a compelling example where the responses of neurons in the central nervous system prompted exploration that led to a candidate algorithm for performing a useful engineering task.

Despite the similarities in top weight combinations and tuning curves, it is an open question as to how a firing rate tuning curve might effectively perform local contrast enhancement. From the perspective of what contrast tuning could be doing to the brain's representation of the signal, a simple explanation might be that contrast tuning effectively skews the neuronal representation of the stimulus contrasts. The majority of neurons, which are un-tuned to contrast, could be rate-coding spectral contrast information with a maximizing information capacity strategy (Laughlin, 1981), such that their spiking activity produces a representation of spectral contrast distribution in the sensory environment. The tuned neurons could use this representation to skew the contrast distribution toward higher contrasts, similar to what happens artificially with the best flat weighting scheme. This could happen if the shape of the rate function of the tuned neurons relative to the input contrast distribution shape represented how much of the respective contrast components are passed through to successive levels in the neuronal network. So, if after some normalization (e.g., relative to the maximum firing rate), the firing rate function matched the input distribution, faithful reproduction could occur. Whereas if the rate deviates lower for low contrasts and higher for high contrasts, the effective output distribution may be closer to what occurs in simulation with the best flat weighting.

The design and implementation of the contrast-shaping signal processing array draws inspiration from neurophysiological findings, although the computational components are not intended to model the physiological implementation of the underlying algorithms. Coupling engineering approaches directly with neurophysiological findings represents a powerful tool for devising new, effective algorithms for use in signal processing. Such a strategy of biologically-inspired engineering derived from cochlear physiology has been quite successful in designing devices to alleviate hearing loss. A broader application of these principles to central nervous system physiology may yield further improvements in these devices, extending analytical results demonstrating that modeling of auditory cortex neuronal responses can be useful for estimating speech intelligibility in normal subjects (Chi et al., 1999).

### Human performance with contrast shaping

Cochlear implantees employing their own implant devices and performing a hearing-in-noise test (HINT) exhibited improved noisy speech intelligibility under the uniform weighting contrast-shaping manipulation. Subjects reported subjectively that these signals sounded much better than the unprocessed noisy signals. Although contrast shaping was not compared directly with other noise-reduction strategies, it appears to be a promising candidate for assisting cochlear implant wearers in noisy situations.

Effective noise-reduction strategies in theoretical tests often show little measurable benefit to human noisy speech intelligibility for non-implantees, even for individuals with considerable hearing loss and even when listeners subjectively indicate a preference for the noise-reduction (Kuk et al., 1990; Moore, 2003). It is possible that real-world tests demonstrate little intelligibility improvement for most individuals because native neuronal noise-reduction circuitry normally does such a good job that trying to improve upon it with an engineered system generally fails to provide additional capabilities (Trine and Tasell, 2002). Even if the above is true, however, noise-reduction strategies could be particularly useful in other situations, such as (1) in subjects such as cochlear implantees, whose native noise-reduction circuitry may not be functioning normally because of the dramatic alteration in signal input relative to normal (Hochberg et al., 1992; Weiss, 1993; Dorman and Loizou, 1996; Loizou and Poroy, 2001; Henry and Turner, 2003; Turner et al., 2004; Henry et al., 2005); (2) in situations where no humans are listening, such as automatic speech recognition (Virag, 1999); or (3) to reduce the effort of understanding speech in noise, even if maximal listening effort does not yield improved speech recognition (Kuk et al., 1990; Moore, 2003; Sarampalis et al., 2006).

## Author contributions

NK, PW, and DB designed the computational model and performed analyses. All authors designed the tests in cochlear implantees. LD conducted the tests in cochlear implantees. NK and DB wrote the paper.

### Conflict of interest statement

The authors declare that the research was conducted in the absence of any commercial or financial relationships that could be construed as a potential conflict of interest.
